# A Rank-Based Sequence Aligner with Applications in Phylogenetic Analysis

**DOI:** 10.1371/journal.pone.0104006

**Published:** 2014-08-18

**Authors:** Liviu P. Dinu, Radu Tudor Ionescu, Alexandru I. Tomescu

**Affiliations:** 1 Faculty of Mathematics and Computer Science, University of Bucharest, Bucharest, Romania; 2 Personal Genetics, Bucharest, Romania; 3 Helsinki Institute for Information Technology HIIT, Department of Computer Science, University of Helsinki, Helsinki, Finland; University of California, San Francisco, United States of America

## Abstract

Recent tools for aligning short DNA reads have been designed to optimize the trade-off between correctness and speed. This paper introduces a method for assigning a set of short DNA reads to a reference genome, under Local Rank Distance (LRD). The rank-based aligner proposed in this work aims to improve correctness over speed. However, some indexing strategies to speed up the aligner are also investigated. The LRD aligner is improved in terms of speed by storing 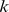-mer positions in a hash table for each read. Another improvement, that produces an approximate LRD aligner, is to consider only the positions in the reference that are likely to represent a good positional match of the read. The proposed aligner is evaluated and compared to other state of the art alignment tools in several experiments. A set of experiments are conducted to determine the precision and the recall of the proposed aligner, in the presence of contaminated reads. In another set of experiments, the proposed aligner is used to find the order, the family, or the species of a new (or unknown) organism, given only a set of short Next-Generation Sequencing DNA reads. The empirical results show that the aligner proposed in this work is highly accurate from a biological point of view. Compared to the other evaluated tools, the LRD aligner has the important advantage of being very accurate even for a very low base coverage. Thus, the LRD aligner can be considered as a good alternative to standard alignment tools, especially when the accuracy of the aligner is of high importance. Source code and UNIX binaries of the aligner are freely available for future development and use at http://lrd.herokuapp.com/aligners. The software is implemented in C++ and Java, being supported on UNIX and MS Windows.

## Introduction

Novel high-throughput sequencing technologies generate up to several millions of short DNA reads (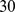 to 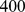 nucleotides long) from random locations in the genome. Putting together these reads into a coherent whole is a significant computational challenge. The first and most expensive step of this process is aligning each read to a known reference genome. Recently, many tools designed to align short reads have been proposed [1]. Sequence alignment tools are designed to optimize the trade-off between correctness and speed, usually sacrificing correctness over speed. This leaves room for new tools for sequence alignment that can better satisfy one of (or both) the two needs, namely efficiency and accuracy. With broad applications from phylogenetic analysis to finding motifs or common patterns in a set of given DNA sequences, new alignment tools are of great interest for the entire community of computational biology researchers.

This paper proposes a method for assigning a set of short DNA reads to a reference genome, under Local Rank Distance (LRD) [2]. Local Rank Distance is an extension of rank distance [3] that is designed to work on overlapping 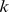-mers instead of single characters as rank distance. Despite the fact that LRD was only recently introduced, it has already demonstrated its performance in phylogenetic analysis [2] and native language identification [4].

The rank-based sequence aligner works as follows. Given a set of reads that need to be aligned against a reference genome, the aligner determines the position of each read in the reference genome that gives the minimum Local Rank Distance. The proposed aligner will be referred to as the *LRD aligner* through the rest of this paper. Some strategies of optimizing the search for the best positions of reads are also proposed and investigated. The LRD aligner is improved in terms of speed by storing 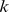-mer positions in a hash table for each read. An approximate LRD aligner that works even faster is obtained through the following strategy. The approximate aligner considers only the positions in the reference that are likely to give the minimum distance, by previously counting the number of 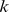-mers from the read that can be found at every position in the reference.

The LRD sequence aligner is designed to work with genomic data produced by Next-Generation Sequencing technologies. These high-throughput technologies are able to produce up to 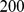 million DNA reads of length between 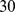 and 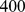 base pairs in a single experiment. Despite this abundance of reads, their short length makes the problem of assembling them into the originating genome a difficult one in practice. Therefore, methods for finding the class, the order, the family or even the species of an unknown organism, given only a set of short Next-Generation Sequencing DNA reads originating from its genome, are of interest. A method that can be used to solve this phylogenetic analysis task is proposed in this work. The method works as follows: given a collection 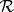 of short DNA reads, and a collection 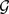 of genomes, it finds the genome 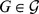 that gives a minimum score. This method serves two purposes. First, the method can be used to determine the place of an individual in a phylogenetic tree, by finding the most similar organism in the phylogenetic tree. This can be achieved by using only a set of short DNA reads originating from the genome of the new individual. Second, the method is used to evaluate the performance level of the rank-based aligner and to compare it with other state of the art alignment tools, such as BWA [5], BOWTIE [6], or BLAST [7]. Experimental results on simulated reads were obtained under two scenarios: low and high error rate. In the former scenario, all the aligners besides BWA have full precision. In the latter scenario, the LRD aligner is the only one that attains full precision. It seems that the LRD aligner gives the most accurate results, while being more computationally expensive than the other aligners.

A set of experiments are conducted to determine the precision and the recall of the proposed LRD aligner, in the presence of contaminated reads. The task is to align reads sampled from several mammals on the human mitochondrial DNA sequence genome. The goal is to maximize the number of aligned reads sampled from the human genome (true positives), and to minimize the number of aligned reads sampled from the other mammals (false positives). Again, the LRD aligner seems to have the best performance, followed closely by BOWTIE and BLAST.

The proposed aligner is also tested on three human vibrio pathogens with results that point towards the same conclusion of [8], [9]. In all the experiments presented in this work, the rank-based aligner shows results that are better than the state of the art alignment tools, in terms of accuracy. The results obtained in this work can be considered as a strong argument in favor of using rank-based distance measures for computational biology tasks, in order to obtain results that are more accurate from a biological point of view.

It is important to point out that the main focus of the experiments is on the alignment accuracy of the aligner based on LRD. Therefore, the simple strategy of assigning each read to the genomic sequence with the best LRD distance was used. However, in other biological problems, these alignments can be fed to other more elaborate methods. For example, in profiling bacterial species from a metagenomics sample, various tools, such as the MG-RAST server [10], MEGAN [11] and metaBEETL [12], align the reads to a reference taxonomy, but report as hit the Lowest Common Ancestor node of a set of significant hits in this taxonomic tree.

### Related Work

#### Similarity Measures Between Genomes

Since most DNA variations between organisms of the same species consist of point mutations like single nucleotide polymorphisms, or small insertions or deletions, edit distance is the standard string measure in many biomedical analyses, such as the detection of genomic variation, genome assembly [13], identification and quantification of RNA transcripts [14]–[16], identification of transcription factor binding sites [17], or methylation patterns [18].

In the case of genomic sequences coming from different related species, other mutations are present, such as reversals [19], transpositions [20], translocations [21], fissions and fusions [22]. For this reason, there have been a series of different proposals of similarity between entire genomes, including rearrangement distance [23], 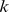-break rearrangements [24], edit distance with block operations [25].

Some of the other popular distance measures for recent computational biology techniques are the Hamming distance [26], [27] and the Kendall-tau distance [28], among others [29]. Rank distance [3] is another such measure of similarity, having low computational complexity, but high significance in phylogenetic analysis [30], [31] and in finding common patterns in DNA sequences [32].

#### Sequence Aligners

One of the most widely used computational biology programs is BLAST [7]. Compared to the previously developed techniques based on dynamic programming [33], BLAST increases the speed of alignment by reducing the search space. An interesting remark is that BLAST calculates the statistical significance for each sequence alignment result.

While BLAST remains an essential tool for biologists, the vast amount of data produced by the high-throughput sequencing technologies led to the development of faster and more accurate sequence aligners. Recently, many tools designed to align short reads have been proposed [1]. The main efforts in the design of such tools are on improving speed and correctness. Fast tools are needed to keep the pace with data production, while the number of correctly placed reads is maximized. Usually tools sacrifice correctness over speed, allowing only few mismatches between the reads and the reference genome. Tools that optimize such trade-off are BOWTIE [6] and BWA [5]. Both the BWA and the BOWTIE aligners work under the edit distance, and they use the Burrows-Wheeler Transform to efficiently align short reads against a large reference sequence, allowing mismatches and gaps. The BOWTIE2 aligner [34] combines the full-text minute index with the flexibility of hardware-accelerated dynamic programming algorithms to achieve both speed and accuracy.

The BFAST [35] tool moves towards favoring correctness over speed, allowing alignments with a high number of mismatches and indels. Another accurate tool able to align reads in the presence of extensive polymorphisms, high error rates and small indels, is rNA [27]. The experiments performed in [27] give an idea about the different approaches of such tools for optimizing the trade-off between correctness and speed. For example, in one experiment BWA is 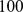 times faster than BFAST, while losing about 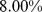 in terms of accuracy.

## Results

### Data Sets

To evaluate the aligners proposed in this work, several experiments are conducted on two data sets of genome sequences. The first data set contains mitochondrial DNA sequence genomes of 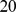 mammals. The genomes are available for download in the EMBL database (http://www.ebi.ac.uk/ena/) using the accession numbers given in Table 1. They belong to the following biological orders: Primates, Perissodactylae, Cetartiodactylae, Rodentia, Carnivora.

**Table 1:** The 20 mammals from the EMBL database used in the phylogenetic experiments. The accession number is given on the last column.

Mammal	Latin Name	Accession No.
human	*Homo sapiens*	V00662
common chimpanzee	*Pan troglodytes*	D38116
pigmy chimpanzee	*Pan paniscus*	D38113
gorilla	*Gorilla gorilla*	D38114
orangutan	*Pongo pygmaeus*	D38115
Sumatran orangutan	*Pongo pygmaeus abelii*	X97707
gibbon	*Hylobates lar*	X99256
horse	*Equus caballus*	X79547
donkey	*Equus asinus*	X97337
Indian rhinoceros	*Rhinoceros unicornis*	X97336
white rhinoceros	*Ceratotherium simum*	Y07726
harbor seal	*Phoca vitulina*	X63726
gray seal	*Halichoerus grypus*	X72004
cat	*Felis catus*	U20753
fin whale	*Balaenoptera physalus*	X61145
blue whale	*Balaenoptera musculus*	X72204
cow	*Bos taurus*	V00654
sheep	*Ovis aries*	AF010406
rat	*Rattus norvegicus*	X14848
mouse	*Mus musculus*	V00711

Mitochondrial DNA (mtDNA) is the DNA located in organelles called mitochondria. The DNA sequence of mtDNA has been determined from a large number of organisms and individuals, and the comparison of those DNA sequences represents a mainstay of phylogenetics, in that it allows biologists to elucidate the evolutionary relationships among species. In mammals, each double-stranded circular mtDNA molecule consists of 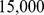 to 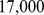 base pairs. DNA from two individuals of the same species differs by only 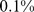. This means, for example, that mtDNA from two different humans differs by less than 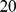 base pairs. Because this small difference cannot affect the study, the experiments are conducted using a single mtDNA sequence for each mammal.

The second data set contains chromosomal DNA sequence genomes of three vibrio pathogens available in the NCBI database (http://www.ncbi.nlm.nih.gov): *Vibrio vulnificus* YJ106, *Vibrio parahaemolyticus* RIMD 2210633, and *Vibrio cholerae* El Tor N16961. The genomes of these three organisms consist of two circular chromosomes. Additional information about these chromosomes, including accession number and size (given in Megabase pairs), is given in Table 2. The genomic sequences of these vibrio species have been revealed by different studies [9], [36], [37]. Several studies report that Vibrio vulnificus shares morphological and biochemical characteristics with other human vibrio pathogens, including Vibrio cholerae and Vibrio parahaemolyticus [8], [9].

**Table 2:** The genomic sequence information of three vibrio pathogens consisting of two circular chromosomes.

Species	Chromosome	Accession No.	Size (Mbp)
*V. vulnificus* YJ016	I (VV1)	NC_005139	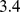
*V. vulnificus* YJ016	II (VV2)	NC_005140	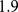
*V. parahaemolyticus* RIMD 2210633	I (VP1)	NC_004603	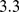
*V. parahaemolyticus* RIMD 2210633	II (VP2)	NC_004605	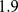
*V. cholerae* El Tor N16961	I (VC1)	NC_002505	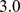
*V. cholerae* El Tor N16961	II (VC2)	NC_002506	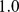

### Alignment in the Presence of Contaminated Reads

In this experiment, reads sampled from the genomes of several mammals are aligned on the human mtDNA sequence genome. The reads were simulated with the *wgsim* tool [38], using the default parameters. More precisely, the reads were generated using an error rate of 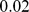, a mutation rate of 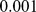, a fraction of indels of 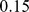 (out of the total number of mutations) and a probability of extending an indel of 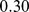.

The LRD aligner is compared to the BWA, the BOWTIE2 and the BLAST aligners, under two different scenarios. In the first scenario, 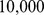 contaminated reads are sampled from the orangutan genome. In the second scenario, 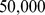 contaminated reads are sampled from 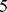 mammals, namely the orangutan, the blue whale, the harbor seal, the donkey, and the house mouse. There are actually 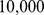 reads sampled from each of the 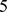 mammals. In both scenarios 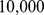 reads simulated from the human genome are included. The simulated reads are always 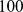 bases long. The goal is to maximize the number of aligned reads sampled from the human genome (true positives), and to minimize the number of aligned reads from the other mammals (false positives). Unlike the other experiments presented in this paper, reverse complement reads were not included in this experiment. However, it is important to mention that the aligners are dealing with a hard task, since the contaminated reads were sampled only from organisms that are in the same class as the human. It may be that contaminated reads from other species that are not in the Mammalia class (such as viruses, for example) can be identified and discarded more easily.

The parameters of the aligners were adjusted as described next. For the BOWTIE2 aligner, two variants are evaluated. The first one uses the *local* and the *very-sensitive-local* options. The second variant uses the *end-to-end* and the *very-sensitive* options. For the BLAST aligner, the *megablast* option is used. Two variants of the LRD aligner based on 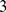-mers and a maximum offset between paired 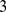-mers of 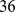 are also evaluated. One is based on the exact search algorithm, while the other one uses the approximate algorithm based on hash tables that runs much faster.

To evaluate and compare the aligners, the precision and recall curve is used. Note that the precision is given by the proportion of aligned reads that are positive, while the recall is given by the proportion of true positive reads that are aligned. In order to obtain the precision-recall curve for each aligner, the idea is to vary the threshold that gives the maximum distance allowed for an aligned read. In the case of the BWA and the BOWTIE aligners, the edit distance threshold takes values from 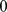 to 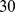. The score of the BLAST aligner ranges from 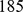 to 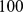. The LRD threshold takes values from 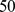 to 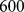, for both variants of the LRD aligner. Higher precision is obtained for lower distance thresholds, while higher recall is obtained for higher distance thresholds. The only aligner that works the other way around, and gives higher precision for higher scores, and higher recall for lower scores, is the BLAST aligner.

Several statistical measures, such as the Area Under the ROC Curve (AUC), the 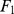 measure, and the 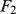 measure, are also presented in order to better compare the aligners. The ROC curve plots the fraction of true positive reads versus the fraction of false positive reads, at various threshold settings. The AUC score represents the area under the ROC curve. The 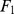 measure (also known as the 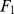 score) can be interpreted as a weighted average of the precision and recall at a certain distance threshold. The 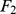 measure is similar to the 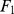 measure, only that it weights recall higher than precision. For each aligner, the highest 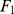 and 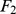 scores can indicate the thresholds that give a good trade-off between precision and recall. The 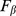 measure is computed as follows: 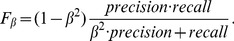
(1)


The 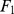 and the 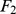 scores are immediately obtained from Equation 1, by replacing 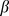 with 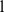 and 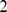, respectively.

#### Human versus Orangutan Experiment

In this experiment, there are 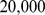 reads to be aligned on the human mtDNA sequence. Half of them are sampled from the same human mitochondrial genome, while the other half are sampled from the orangutan mitochondrial genome. Thus, the contamination rate is 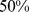.

**Figure 1:**
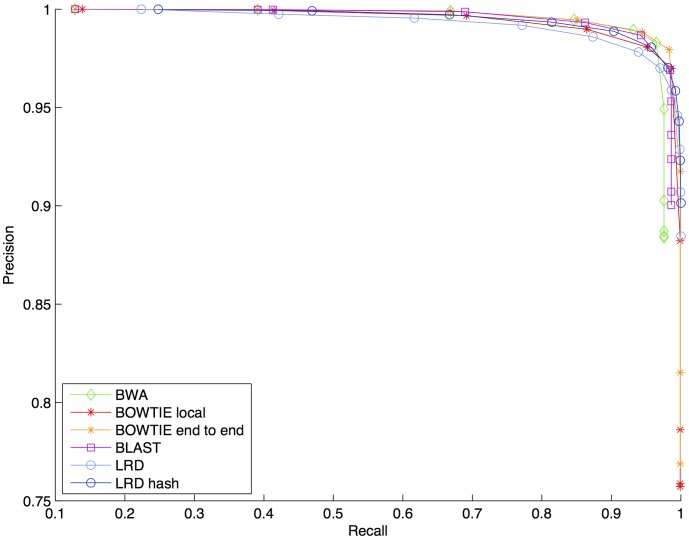
The precision-recall curves of the state of the art aligners versus the precision-recall curve of the two LRD aligners, when 10,000 contaminated reads of length 100 from the orangutan are included. The two variants of the BOWTIE aligner are based on local and global alignment, respectively. The LRD aligner based on hash tables is a fast approximate version of the original LRD aligner.

The precision-recall curves of the BWA, the BOWTIE, and the BLAST aligners together with the precision-recall curves of the two variants of the LRD aligner are presented in Figure 1. By analyzing Figure 1, it can be observed that the aligners obtain roughly similar results in terms of precision and recall. To better assess the performance of the evaluated aligners, the AUC measure and the best 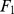 and 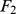 scores for each aligner are presented in Table 3. In terms of the AUC, the BOWTIE and the LRD aligners attain the best results, while the other aligners fall behind. In terms of the 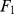 measure, the BOWTIE aligner seems to be slightly better than the LRD aligner, while in terms of the 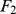 measure, the LRD aligner achieves the best score, followed closely by the BOWTIE aligner. The BLAST aligner comes in third place after the LRD and the BOWTIE aligners. The results of the BWA aligner are also not too far from the other top scoring aligners.

**Table 3:** Several statistics of the state of the art aligners versus the LRD aligner, when 10,000 contaminated reads of length 100 sampled from the orangutan genome are included.

Aligner	AUC	Best 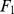 Score	Best 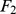 Score
BWA	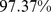	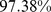	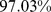
BOWTIE local	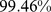	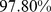	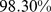
BOWTIE end-to-end	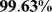	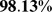	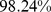
BLAST	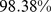	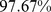	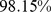
LRD aligner	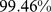	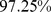	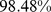
Hash LRD aligner	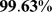	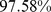	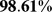

The AUC is computed from the ROC curve, while the best 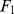 and 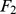 measures where computed using different points on the precision-recall curve. The 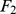 measure puts a higher weight on recall.

The results presented in Figure 1 indicate that all the aligners obtain a good trade-off between precision and recall. Indeed, all of them are able to align more than 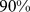 of the human reads with a precision that is higher than 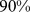. For instance, the hash LRD aligner is able to align 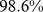 of the humand reads with 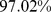 precision. However, it would be interesting to observe how the LRD aligner behaves at the sequence level. For this purpose, some metrics of the reads simulated from the human mitochondrial genome are provided in Table 4. More precisely, the average Hamming distance and the average edit distance of the human reads that are mapped to the human genome (true positives) are reported at different precision and recall levels. In the same time, the average Hamming distance and the average edit distance of the human reads that are not mapped to the human genome (false negatives) are also reported. Perhaps it would be more interesting to give the average number of errors and mutations in the true positive reads versus the average number of errors and mutations in the false negative reads. Unfortunately, the *wgsim* tool does not output these values for the simulated reads. Nevertheless, the simulation tool does output the exact location from which each read was simulated. Therefore, a standard distance can be computed between a simulated read and its corresponding original substring (of 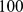 bases) from the human genome, that was used by *wgsim* to generate the read. The Hamming distance and the edit distance together should give some indication of the number of changes in the human reads that are not mapped to the human genome. It can be observed that for each LRD threshold presented in Table 4, the average Hamming distance of the mapped reads is always less than the average Hamming distance of the false negative reads. The same statement is also valid for the edit distance. For both distance measures, the difference between the average distance of true positives and the average distance of false negatives is not very high. Basically, only a few more bases are different from the source substring for the false negatives compared to the true positives. The highest difference is reported for the LRD threshold of 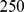. Table 4 shows that, on average, the reads that are mapped to the genome have less errors and mutations than the reads that are not mapped. However, the difference is not significant, since the false negative reads have at most 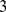 more errors, on average, than the mapped reads. An interesting remark is that the LRD aligner accepts more and more errors and mutations in the aligned reads as the LRD threshold increases, but even with the highest threshold of 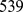 that gives 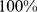 recall rate, the precision of the hash LRD aligner is still very high (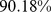). In other words, the LRD aligner does a good job at discarding most of the reads simulated from the orangutan genome (true negatives), while mapping all the human reads, even those with higher error rates.

**Table 4:** Metrics of the human reads mapped to the human mitochondrial genome (true positives) by the hash LRD aligner versus the human reads that are not mapped to the genome (false negatives).

LRD	Precision	Recall	TP	FN	TP Ham.	FN Ham.	TP edit	FN edit
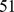	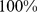	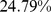	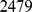	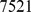	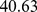	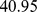	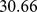	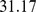
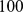	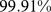	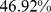	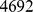	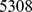	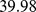	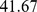	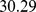	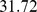
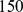	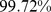	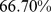	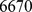	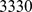	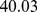	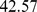	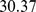	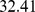
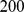	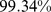	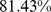	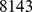	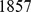	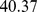	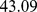	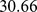	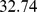
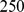	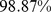	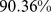	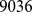	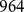	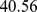	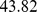	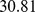	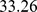
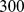	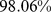	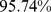	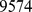	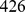	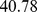	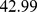	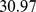	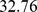
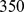	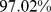	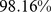	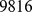	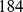	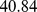	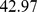	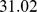	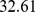
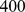	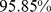	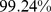	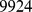	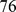	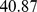	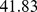	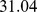	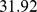
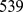	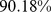	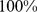	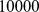	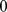	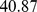	-	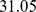	-

The average Hamming distance and the average edit distance are reported for true positive (TP) and false negative (FN) reads, respectively. The average distances are given for several points on the precision-recall curve of the hash LRD aligner, going from 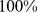 precision to 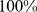 recall. The points are obtained by varying the LRD threshold from 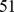 to 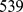.

#### Human versus Five Mammals Experiment

In this second experiment, there are 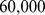 reads to be aligned on the human mtDNA sequence. Only 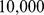 reads are actually sampled from the same human genome. The 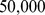 contaminated reads where sampled from 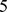 different mammals. The mammals where chosen to represent the 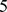 orders available in the first data set: Primates, Perissodactylae, Cetartiodactylae, Rodentia, and Carnivora. The contamination rate of 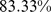 is much higher than in the previous scenario.

**Figure 2:**
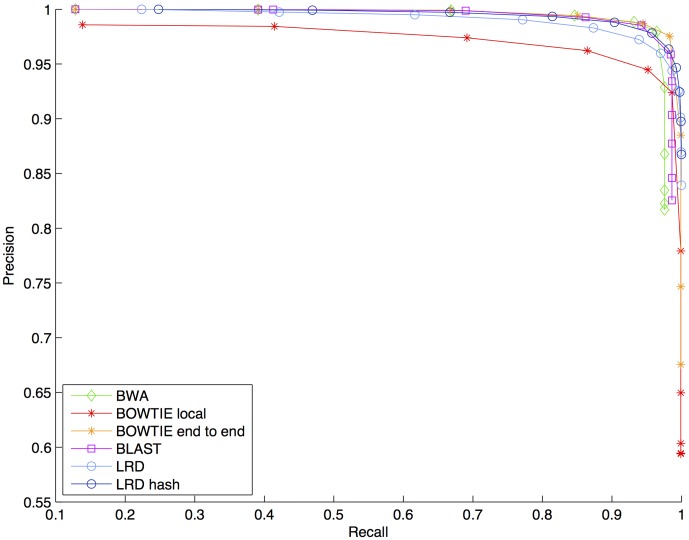
The precision-recall curves of the state of the art aligners versus the precision-recall curves of the two LRD aligners, when 50,000 contaminated reads of length 100 from 5 mammals are included. The two variants of the BOWTIE aligner are based on local and global alignment, respectively. The LRD aligner based on hash tables is a fast approximate version of the original LRD aligner.

The precision-recall curves of the BWA, the BOWTIE, and the BLAST aligners versus the precision-recall curve of the two variants of the LRD aligner are presented in Figure 2. Among the evaluated aligners, the BOWTIE local aligner has the lowest results in terms of precision and recall. Figure 2 seems to indicate that the LRD, the BLAST, and the BOWTIE end-to-end aligner give fairly similar results.

To make a better distinction between the aligners, the AUC measure and the best 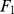 and 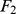 scores for each aligner are presented in Table 5. The results presented in Table 5, indicate that the LRD aligner achieves the best AUC score, followed closely by the BOWTIE end-to-end aligner. As in the previous experiment, the BOWTIE aligner attains the highest 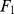 score, while the LRD aligner attains the highest 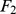 score. The BLAST aligner falls in third place.

**Table 5:** Several statistics of the state of the art aligners versus the LRD aligner, when 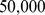 contaminated reads of length 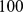 sampled from the genomes of 5 mammals are included.

Aligner	AUC	Best 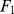 Score	Best 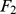 Score
BWA	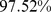	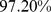	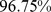
BOWTIE local	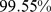	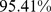	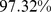
BOWTIE end-to-end	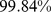	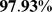	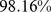
BLAST	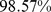	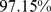	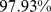
LRD aligner	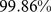	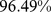	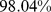
Hash LRD aligner	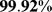	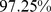	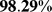

The AUC is computed from the ROC curve, while the best 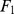 and 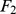 measures where computed using different points on the precision-recall curve. The 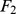 measure puts a higher weight on recall.

An advantage of the LRD aligner is that it is the most flexible aligner in terms of precision and recall. The aligner proposed in this work is the only aligner that can be adjusted to go from 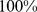 precision to 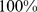 recall. Even if the other state of the art aligners do reach full recall, it is interesting to show the best recall that can be obtain by each one. The BWA aligner reaches a maximum recall of 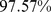, while the BLAST aligner reaches a maximum recall of 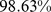. Both variants of the BOWTIE aligner go up to 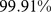 recall. As mentioned before, the maximum recall obtained by the LRD aligner is 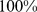.

Another interesting statistics is the recall when 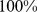 precision is achieved. The recall at best precision is recorded in two scenarios. In the first scenario, only the contaminated reads from the orangutan are included, while in the second scenario, the rest of 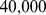 contaminated reads from all the other mammals, besides the orangutan, are included. Since the orangutan and the human belong to the Primates order, the first scenario is more difficult.

The recall at best precision for each aligner evaluated in the first scenario is given in Table 6. When 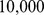 contaminated reads sampled from the orangutan genome are used, it seems that the LRD aligner obtains the highest recall at 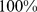 precision. The LRD aligner is roughly 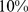 higher than the state of the art aligners, which give similar recall values to each other.

**Table 6:** The recall at best precision of the state of the art aligners versus the LRD aligner, when 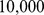 contaminated reads of length 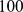 sampled from the orangutan genome are included.

Aligner	Recall at Best Precision	Best precision
BLAST	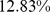	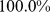
BWA	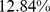	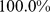
BOWTIE end-to-end	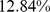	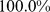
BOWTIE local	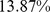	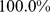
LRD aligner	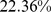	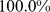
Hash LRD aligner	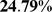	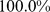

The recall at best precision for each aligner evaluated in the second scenario is given in Table 7. This time, the recall at 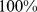 precision for each aligner is much higher than in the first scenario. This indicates that if contaminated reads do not belong to an organism that is closely related to the human, the tools are able to align most of the true positive reads with 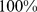 precision. Again, the best aligner is the LRD aligner based on the hash tables implementation. It attains a recall of 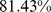, being roughly 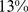 better than most of the state of the art aligners. In the second scenario, it seems that the BOWTIE local aligner falls very far behind the other alignment tools.

**Table 7:** The recall at best precision of the state of the art aligners versus the LRD aligner, when 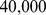 contaminated reads of length 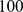 sampled from the blue whale, the harbor seal, the donkey, and the house mouse genomes are included, respectively.

Aligner	Recall at Best Precision	Best precision
BLAST	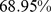	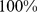
BWA	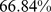	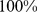
BOWTIE end-to-end	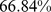	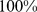
BOWTIE local	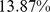	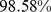
LRD aligner	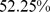	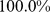
Hash LRD aligner	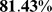	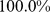

Overall, the LRD aligner seems to be the best tool among the evaluated aligners, in the presence of contaminated reads. It is closely followed by the BOWTIE end-to-end aligner. The high accuracy of the LRD aligner comes with the cost of being the slowest one among the evaluated aligners.

### Clustering an Unknown Organism

The rank-based aligner is evaluated in the context of finding a solution for the task of clustering a new (or unknown) organism, given only a set of short Next-Generation Sequencing DNA reads. More precisely, the task is to find the order, the family, or the species of the unknown organism, without having to sequence its genome first, by aligning its reads into several genomes in order to obtain the nearest neighbor species (or the most similar species). The LRD aligner is compared to the BWA, the BOWTIE2 and the BLAST aligners. In the case of the BOWTIE2 aligner, two variants are evaluated, one based on local alignment and the other based on global alignment. The LRD aligner is based on 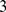-mers with a maximum offset between paired 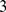-mers of 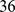. A maximum distance threshold of 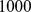 was used in the case on the LRD aligner. The distance threshold for the LRD aligner was adjusted in order to allow more reads to be aligned, especially for the mammals that are more distantly related, more precisely, that are not from the same order. The approximate hash LRD aligner achieves similar results to the basic LRD aligner, when it aligns reads only in the positions that have at most 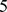 similar 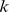-mers less than the maximum number of 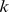-mers from the read that can be found at any given position in the reference sequence. For this reason, only the results of the approximate LRD aligner are reported in the following experiments.

One by one, each of the 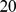 mammalian genomes from the EMBL database will be considered to be unknown for the purpose of this experiment. The unknown individual will be represented by a set .. of short DNA reads randomly sampled from its genome. The task is to find the most similar individual (or species) from the remaining 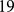 individuals, for each unknown individual. In order to solve the task, the collection 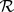 of reads (that represents an unknown individual) is aligned on each of the 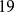 genomes from the collection 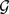 of genomes. Reads are aligned under a maximum distance threshold. Thus, only a subset 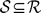 of reads is aligned on each genome. An alignment score is computed for each genome in order to obtain the most similar individual. The score is given by the average minimum distances of the reads in 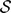 divided by the number of aligned reads. The minimum distance for a specific read is given by the best positional match in the reference genome. Lower scores indicate greater similarity between species, and higher scores indicate a greater dissimilarity between species. The individual (or the species) with the lowest score is considered to be the most similar one. Finally, the unknown organism is considered to be part of the same order as its most similar individual. The unknown individual is correctly clustered if it is indeed a member of the order predicted by the aligner. Thus, the performance of each aligner on this task is determined by the number of correctly clustered unknown individuals. The evaluation procedure can also be described as the leave-one-out cross-validation procedure. It is important to notice that the procedure described above does not generate a partitioning of the data set, but rather assigns a newly discovered (or unknown) organism to a specific cluster in an existing phylogenetic tree. An evaluation tool to obtain this score has also been added to the software package.

An interesting remark is that the tools evaluated on this task align reads under a given maximum distance threshold and, hence, many reads remain unaligned. The distance measure depends on the aligner. While the BWA and the BOWTIE aligners are based on the edit distance, the BLAST aligner uses a score of its own. The rank-based aligner is based on Local Rank Distance. Therefore, the alignment score is obtained by the average distance divided by the number of aligned reads. In other words, a genome with more aligned reads is more likely to be similar to the unknown individual.

The aligners are evaluated and compared under two different scenarios. In both scenarios, reads of 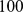 bases long were simulated using the *wgsim* tool [38]. In the first scenario, 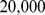 short DNA reads per mitochondrial genome are sampled using the default parameters of the simulation tool. More precisely, the reads were generated using an error rate of 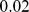, a mutation rate of 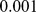, a fraction of indels of 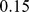 (out of the total number of mutations) and a probability of extending an indel of 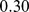. With an average base coverage of 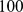, the number of reads should be far than enough to correctly determine the order of unknown organisms. This scenario is designed to simulate a real-world setting where a high number of Next-Generation Sequencing reads is usually available. In the second scenario, only 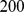 simulated short DNA reads per genome are used in order to make the task harder to solve. The alignment methods should be challenged by the small amount of available reads. The generated reads also have more errors. More precisely, the reads for this second test case were simulated using an error rate of 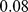, a mutation rate of 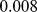, a fraction of indels of 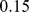 (out of the total number of mutations) and a probability of extending an indel of 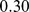. In both test cases, half of the simulated reads from each genome are reverse complements.

#### Real-World Setting Experiment on Mammals

In the first test case, 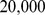 simulated DNA reads of length 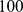 per genome are used, which corresponds to an average base coverage of 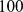. Table 8 compares the results of the LRD aligner with the other state of the art aligners.

**Table 8:** The results for the real-word setting experiment on mammals.

Mammal	Class	BWA	BWA	BLAST	BOWTIE	BOWTIE	LRDa
	(Label)	edit 5	edit 10		local	end-to-end	LRD 1000
cow	Cet (1)	4	9*	2	2	2	2
sheep	Cet (2)	1	9*	1	1	1	1
blue whale	Cet (3)	4	4	4	4	4	4
fin whale	Cet (4)	6*	3	3	3	3	3
cat	Car (5)	9*	9*	7	7	7	6
gray seal	Car (6)	7	7	7	7	7	7
harbor seal	Car (7)	6	6	6	6	6	6
human	Pri (8)	11	11	11	11	11	11
gibbon	Pri (9)	8	2*	8	11	13	11
gorilla	Pri (10)	5*	13	13	11	11	11
p. chimpanzee	Pri (11)	13	13	13	13	13	13
orangutan	Pri (12)	14	14	14	14	14	14
chimpanzee	Pri (13)	11	11	11	11	11	11
S. orangutan	Pri (14)	12	12	12	12	12	12
horse	Per (15)	16	16	16	16	16	16
donkey	Per (16)	15	15	15	15	15	15
I. rhinoceros	Per (17)	18	18	18	18	18	18
w. rhinoceros	Per (18)	17	17	17	17	17	17
mouse	Rod (19)	20	17*	20	20	20	20
rat	Rod (20)	19	17*	19	19	19	19
**Accuracy**		17/20	14/20	20/20	20/20	20/20	20/20

The results of clustering unknown organisms using the BWA aligner, the BLAST aligner, the BOWTIE aligner and the LRD aligner are presented on columns, respectively. Mammals are labeled with numbers from 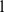 to 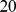, given on the second column. The label of the closest species obtained by each aligner is reported for each mammal. Incorrectly clustered mammals are marked in bold and with an asterisk. Classes are actually 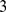-letter prefixes of order names. Unknown organisms are represented by 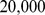 reads of length 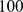 simulated from the original genomes. Half of the reads are reverse complements.

The BWA aligns only the reads that fall under a certain edit distance threshold. The BWA aligner based on the default threshold 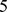 is listed in Table 8 under the name of *BWA edit 5*. Another BWA aligner with a threshold of 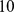 was used in the experiments. Since the latter one aligns more reads, it should be able to give more accurate results than the default BWA aligner.

In this scenario, it seems that the BLAST, the BOWTIE and the LRD aligners achieve perfect results. More precisely, they are all able to identify the most similar individual as being part of the same order as the unknown organism, for the entire set of 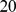 mammals. On the other hand, the BWA edit 5 aligner is only able to predict the correct order for 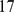 out of 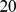 mammals. It clusters the cat as Primates, and the fin whale and the gorilla as part of the Carnivora order. The BWA edit 10 aligner works even worse, correctly predicting the order for 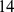 mammals.

It is interesting to observe that all the methods are usually able to determine not only the correct order, but also the most similar species in the group. For example, the horse is always clustered near the donkey, rather than the Indian or the white rhinoceros, despite the fact that they are all members of the same order, namely Perissodactylae. The same situation can be observed in the case and the gray seal, which is always considered to be most similar with the harbor seal rather than the other member of the Carnivora order, namely the cat.

The empirical results show that, with the exception of the BWA aligner, all the other methods work very well. This also demonstrates that the evaluation procedure gives a relevant measure of similarity between a set of reads and a reference genome, that can be used for solving the task of clustering unknown organisms.

#### Hard Setting Experiment on Mammals

The first test case is not enough to make a clear distinction between the compared methods, with respect to the accuracy and the biological relevance. To better assess the performance levels of these aligners, another experiment is conducted using only 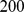 short DNA reads of length 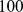 per genome. As described above, the reads also contain more errors and mutations than in the previous test case.

The results of the state of the art aligners together with the results of the LRD aligner are shown in Table 9. Compared to the previous scenario, the results of the state of the art aligners are much lower this time. The BWA aligners predict the correct order for 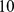 and 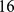 mammals, respectively. Unlike the previous test case, the BWA edit 10 aligner works better than the BWA edit 5 aligner, probably because it is able to align more reads with high error and mutation rates. The BOWTIE aligners obtain results that are roughly similar to the results of the BWA aligners. The BOWTIE local aligner predicts the right order for 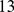 out of 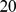 mammals, while the BOWTIE end-to-end aligner is able to correctly cluster two more mammals, reaching a total of 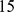 correctly clustered mammals. The BLAST aligner works fairly well, predicting the correct order for 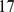 mammals. It wrongly predicts the order for the cat and for the two members of the Rodentia order, namely the house mouse and the rat. It seems that all the aligners, besides the LRD aligner, have trouble predicting the right order for the Rodentia members. On the other hand, it seems that the aligners find it very easy to predict the correct order for the Primates. Finally, the LRD aligner is able to predict the correct class for the entire set of mammals. The LRD aligner seems to be more robust to high error and mutation rates, as it achieves the best results among all the evaluated aligners.

**Table 9:** The results for the hard setting experiment on mammals.

Mammal	Class	BWA	BWA	BLAST	BOWTIE	BOWTIE	LRDa
	(Label)	edit 5	edit 10		local	end-to-end	LRD 1000
cow	Cet (1)	*	2	2	19*	2	2
sheep	Cet (2)	*	5*	1	1	12*	1
blue whale	Cet (3)	*	4	4	12*	4	4
fin whale	Cet (4)	3	1	3	3	3	3
cat	Car (5)	*	*	1*	9*	19*	7
gray seal	Car (6)	7	7	7	7	7	7
harbor seal	Car (7)	6	6	6	6	6	6
human	Pri (8)	*	13	11	11	13	11
gibbon	Pri (9)	*	11	13	16*	14	13
gorilla	Pri (10)	8	11	8	11	8	11
p. chimpanzee	Pri (11)	13	13	13	13	13	13
orangutan	Pri (12)	*	14	14	14	14	14
chimpanzee	Pri (13)	11	11	11	11	11	11
S. orangutan	Pri (14)	12	12	12	12	12	12
horse	Per (15)	16	16	16	16	16	16
donkey	Per (16)	15	15	15	15	15	15
I. rhinoceros	Per (17)	18	18	18	15	12*	18
w. rhinoceros	Per (18)	*	17	17	14*	17	17
mouse	Rod (19)	*	6*	12*	14*	12*	20
rat	Rod (20)	*	*	12*	8*	5*	19
**Accuracy**		10/20	16/20	17/20	13/20	15/20	20/20

The results of clustering unknown organisms using the BWA aligner, the BLAST aligner, the BOWTIE aligner and the LRD aligner are presented on columns, respectively. Mammals are labeled with numbers from 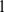 to 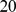, given on the second column. The label of the closest species obtained by each aligner is reported for each mammal. Incorrectly clustered mammals are marked in bold and with an asterisk. Classes are actually 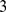-letter prefixes of order names. Unknown organisms are represented by 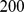 reads of length 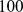 (half of them being reverse complements) simulated from the original genomes, using an error rate of 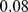 and a mutation rate of 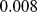.

It is interesting to observe that the BWA with an edit distance threshold of 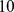 is not able to align any reads at all, for two of the mammals. This is the reason why no similar mammal is found for the cat or for the rat. The same problem occurs in the case of the BWA edit 5 aligner, which is not able to find any similar genomes for 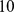 mammals, due to the lack of aligned reads. This problem is likely caused by the high error and mutation rates, that were used to sample the reads from the original genomes. It may be concluded that the BWA aligner is the most fragile aligner with respect to high error and mutation rates.

#### Time Evaluation

The time taken by each aligner to produce the results for the two test cases of the experiment on clustering unknown organisms is shown in Table 10. For both test cases, there are 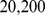 short DNA reads that must be aligned for each mammal on the rest of 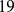 mammalian genomes. In total, each tool must align 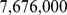 short DNA reads of 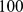 bases long, on a reference mtDNA genome of roughly 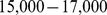 bases. Note that the reference genome is not necessarily always the same, since the reads sampled from a genome are aligned into the remaining 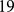 genomes. The time was measured on a computer with Intel Core i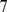 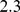 GHz processor and 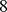 GB of RAM memory using a single Core.

**Table 10:** The running times of the BWA aligner, the BLAST aligner, the BOWTIE aligner and the LRD aligner.

Method	Time
BWA edit 5	3 minutes 14 seconds
BWA edit 10	3 minutes 50 seconds
BOWTIE local	9 minutes 43 seconds
BOWTIE end-to-end	7 minutes 14 seconds
BLAST	30 minutes
LRDa	285 hours
LRDa + hash (C++ implementation)	16 hours 33 minutes
LRDa + hash (Java implementation)	326 minutes

The aligners are compared on the task of aligning 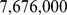 short DNA reads of 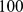 bases long on a reference mtDNA genome of roughly 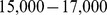 bases. The aligners were evaluated on a computer with Intel Core i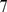 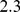 GHz processor and 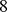 GB of RAM memory using a single Core.

Among the evaluated aligners, the BWA aligner is the fastest one, taking just over 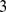 minutes to align all the reads. The BOWTIE2 aligner is also very fast. It takes roughly 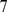 minutes to align the reads when the *local* option is used, and 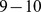 minutes for the *end-to-end* option. The BLAST aligner takes 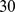 minutes when the *megablast* option is turned on. Finally, the LRD aligner is the slowest one, but it also has the advantage of being the most accurate on all the test cases. The approximate LRD aligner based on the hash optimization implemented in C++ needs 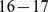 hours to align all the reads. The Java implementation of LRD aligner based on hash tables is roughly 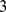 times faster, with a total time of 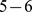 hours. The speed gain of the Java implementation is given by the optimized hash table implementation available in the Java API. It is important to mention that the parameters of the approximate LRD aligner are optimized for accuracy, not for speed. Even so, the approximate hash LRD aligner implemented in Java is roughly 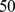 times faster than the basic LRD aligner. The reported time of the approximate hash LRD aligner is comparable to that of the other tools that favor correctness over speed, such as BFAST [35]. Parallel or GPU processing could be used to further reduce the running time of the LRD aligner and to make it run as fast as BOWTIE2 or BLAST.

An important advantage of the LRD aligner is that it obtains very accurate results even for a very low base coverage. For instance, the LRD predicts the correct order for the entire set of 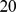 mammals by aligning 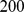 reads per genome (with high error and mutation rates), while the BWA edit 5 aligner is only able to predict the correct order for 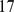 mammals using 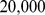 reads per genome (with low error and mutation rates). Considering this fact, the LRD aligner obtains better results than the fastest aligner (BWA) in the same amount of time (roughly 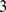 minutes). This being said, the LRD aligner can produce accurate results in an amount of time which is comparable the other state of the art aligners, simply by aligning considerably less reads than the other tools would require.

### Experiment on Vibrio Species

In [9], a comparative study of the *V. vulnificus* YJ106, *V. parahaemolyticus* RIMD 2210633, and *V. cholerae* El Tor N16961 genomes was conducted to compare relative positions of conserved genes and to investigate the movement of genetic materials within and between the two chromosomes of these vibrio species. The study shows that *V. vulnificus* has a higher degree of conservation in gene organization in the two chromosomes relative to *V. parahaemolyticus* rather than to *V. cholerae*. This implies that *V. vulnificus* is closer to *V. parahaemolyticus* than to *V. cholerae* from the evolutionary point of view. This result is also supported by the study of [8], which determines that the block-interchange distance between *V. vulnificus* and *V. parahaemolyticus* is smaller than that between *V. vulnificus* and *V. cholerae*.

The goal of this experiment is to determine if the LRD aligner can achieve similar results to [8], [9], using the evaluation procedure for clustering an unknown organism proposed in this work. Thus the experiment consists of aligning simulated reads from the *V. vulnificus* chromosomes into *V. parahaemolyticus* and *V. cholerae*. It is important to note that three test cases were considered. In the first test case, simulated reads of chromosome VV1 are aligned into VP1 and VC1, respectively. In the second case, simulated reads of chromosome VV2 are aligned into VP2 and VC2, respectively. Finally, the simulated reads from both chromosomes of *V. vulnificus* are aligned into the two chromosomes of *V. parahaemolyticus* on one hand, and into the two chromosomes of *V. cholerae* on the other hand.

In this experiment, reads of 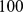 bases long were simulated using the default parameters of the *wgsim* tool [38]. More precisely, the reads were generated using an error rate of 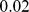, a mutation rate of 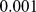, a fraction of indels of 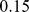 (out of the total number of mutations) and a probability of extending an indel of 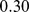. In this experiment, 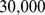 simulated reads per chromosome are used, which corresponds to an average base coverage of 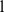. As in the previous experiment, half of the simulated reads from each genome are reverse complements. The LRD aligner is based on 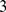-mers with a maximum offset between paired 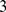-mers of 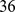. As in the previous experiments, the maximum distance threshold is set to 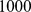.

The scores of simulated reads from *V. vulnificus* chromosomes I and II aligned into *V. parahaemolyticus* and *V. cholerae* using the LRD aligner are shown in Table 11. The empirical results for all the three test cases are presented in this table. Each score is given by the average minimum Local Rank Distances of the aligned reads divided by the number of aligned reads on each genome. The results of the LRD aligner are similar to the results obtained in [8], [9]. More precisely, the score between *V. vulnificus* and *V. parahaemolyticus* is lower than that between *V. vulnificus* and *V. cholerae* for both chromosomes of the three vibrio species. Even if chromosomes I and II are combined, *V. vulnificus* is found to be more similar to *V. parahaemolyticus*.

**Table 11:** The results of the rank-based aligner on vibrio species.

Reads Source	Reference	LRDa Score
VV1	VP1	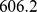
VV1	VC1	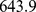
VV2	VP2	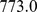
VV2	VC2	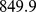
VV1 + VV2	VP1 + VP2	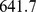
VV1 + VV2	VC1 + VC2	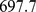

The LRD aligner is based 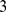-mers, a maximal offset of 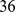, and a LRD threshold of 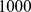. The scores obtained by the LRD aligner for simulated reads of *V. vulnificus* chromosomes I and II aligned into *V. parahaemolyticus* and *V. cholerae* are presented in this table. The first column indicates the source chromosome of the simulated reads. The second column indicates the reference chromosome. The third and fourth columns show the scores of the two aligners computed with the evaluation tool provided in the software package.

Some concern regarding the results obtained in this experiment might be that the results are influenced by the length difference between the reference genomes of *V. parahaemolyticus* and *V. cholerae*. First of all, the difference between the scores obtained by the LRD aligner is much higher than the difference between the lengths of the chromosomes VP1 and VC1. However, the study might be affected by the significant length difference between VP2 and VC2. While the number of simulated reads is fixed, the alignment tool excludes the reads that show a distance that is higher than the maximum threshold of 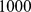. The threshold should remove most of the reads that are aligned by chance, thus giving a score that is not influenced by the longer length of the VP2 chromosome.

## Discussion

The results of the LRD aligner presented in this work are obtained using 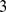-mers and a maximum offset of 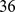. The maximum offset depends on the read length, more precisely it should be less or equal to the read length. The 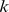-mers length should also be adjusted with regard to the read length. For reads of length 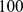, 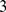-mers are a reasonable choice since the chances of finding matching pairs of 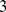-mers between a read and the genome are very high. But even 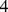-mers and 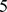-mers work well, especially if the reads and the reference genome belong to the same species. If longer reads are considered for alignment, even longer 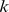-mers can be used for a better accuracy and speed. On the other hand, longer 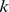-mers are likely to reduce the accuracy of the aligner when the mutation and error rates are high, since the longer is the 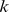-mer the greater is the probability of containing a mutation or error. For instance, if a 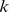-mer contains a point mutation, the 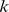-mer will not be matched correctly when LRD is computed. Even if LRD is designed to handle such situations, a carefully chosen 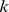-mer length can make the most of the aligner proposed in this work. For instance, the work of [2] shows that LRD can be used with 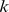-mers ranging from 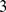 to 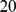 letters. In the phylogenetic analysis of mammals presented in [2], the best results are obtained with 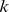-mers ranging from 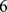 to 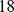 letters. When the LRD aligner is used for a specific application, it is recommended to tune the parameters of the aligner on a validation data set first, by considering the guidelines provided in this work.

Overall, the LRD aligner gives the most accurate results and it seems to be very robust for reads that contain many errors or mutations. However, the accurate results of LRD come with a cost. The time that LRD takes to align the same number of reads is higher than the time of the state of the art aligners evaluated in this paper. Nevertheless, the empirical results presented in this work show that the LRD aligner can produce very accurate results in the same amount of time as the other alignment tools, simply by using a lower base coverage. There is still enough room to speed up the LRD algorithm. By implementing it on GPU, the LRD aligner will be comparable (in terms of time) with the other aligners that favor efficiency over correctness. The LRD aligner can be considered as an useful tool for sequence alignment, being highly accurate from a biological (or evolutionary) point of view.

It is worth mentioning that another aligner based on rank distance (RD) was also proposed and evaluated. Despite the fact that the RD aligner is twice as fast as the BLAST aligner, the results obtained by the RD aligner on the set of experiments presented in this paper were not very convincing in terms of accuracy. More precisely, it seems that the RD is not able to distinguish contaminated reads when a high recall is desired. On the other hand, it was able to identify the order of unknown organisms at a success rate comparable to the state of the art aligners. The RD aligner is included in the software package provided by this work for future development.

**Figure 3:**
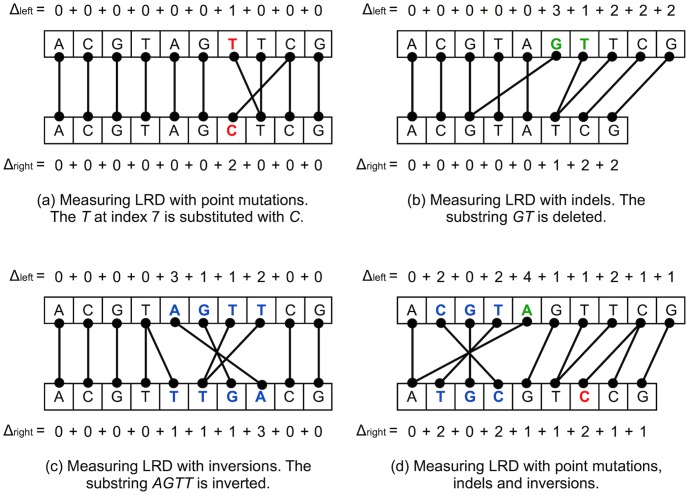
Local Rank Distance computed in the presence of different types of DNA changes such as point mutations, indels and inversions. In the first three cases (a), (b) and (c), a single type of DNA polymorphism is included in the second (bottom) string. The last case (d) shows how LRD measures the differences between the two DNA strings when all the types of DNA changes occur in the second string. The nucleotides affected by changes are marked with bold. To compare the results for the different types of DNA changes, the first string is always the same in all the four cases. Note that in all the four examples, LRD is based on 1-mers. In each case, 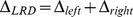.

The results presented in this work can be considered as a strong argument in favor of using Local Rank Distance for computational biology tasks, in order to obtain results that are often more accurate from a biological point of view. Local Rank Distance [2] is related to the rearrangement distance [39]. The rearrangement distance works with indexed 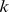-mers and is based on a process of converting a string into another, in a similar fashion to the edit distance. Unlike the edit distance or the rearrangement distance, LRD does not impose such global constraints. Instead, LRD tries to capture only the local changes in DNA. This seems to be more natural from an evolutionary point of view, since changes in DNA, such as point mutations or indels, occur at the local level. Perhaps this is the key insight of why Local Rank Distance should be expected to give more accurate results than the other distance measures. For instance, the edit distance counts the minimum number of operations required to transform one string into the other. It is clear that the actual number of DNA changes that did occur may be higher than the minimum number of operations. The Hamming distance sides with Local Rank Distance regarding the local aspect. However, the Hamming distance is greatly affected by indels. A single character that is inserted (or deleted) into one of the two strings will damage the Hamming distance computation for the rest of string. On the other hand, Local Rank Distance is more robust to changes such as indels or duplications, since it sums up the positional offsets of identical 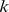-mers. When two DNA sequences are identical, the positional offsets of identical 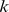-mers sum up to zero. If the two DNA sequences are affected by various types of DNA changes, the positional offsets of identical 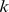-mers increase mostly in the affected DNA regions. Consequently, the Local Rank Distance will be higher, since it finds displaced 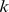-mers. When more point mutations, indels, reversals or other kinds of errors occur in the DNA, LRD will indicate an even higher distance between the DNA sequences. Intuitively, Local Rank Distance reflects the total amount of local changes between two DNA sequences. This intuition can be better observed in Figure 3, which shows how the Local Rank Distance between two DNA sequences changes when one of the two sequences is affected by different types of DNA polymorphisms. Another key insight of why the rank-based approach should work better is that Local Rank Distance can capture very fine differences between strings, unlike the more commonly used edit distance or Hamming distance. More results that support this statement are presented in the empirical study performed in [32], which compares rank distance with Hamming distance and edit distance, respectively.

### Conclusion and Further Work

This paper presented a tool for aligning a set of short DNA reads inside a reference genome, under Local Rank Distance. Several strategies for improving the speed of the LRD aligner were proposed. First of all, the 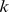-mer positions were stored in a hash table for each read. Second of all, only the positions in the reference that are likely to give the minimum distance were considered, by previously counting the number of 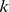-mers from the read that can be found at each position in the reference.

A set of experiments were conducted to assess the performance of the rank-based aligner in the presence of contaminated reads. In another set of experiments, the proposed aligner was used to find a solution for the task of clustering an unknown individual, given only a set of short DNA reads. Compared to the other evaluated tools, the LRD aligner has the important advantage of being very accurate even for a very low base coverage. To conclude, the empirical results showed that the LRD aligner can be considered as a viable alternative to standard alignment tools, since it can often be more accurate. Furthermore, the results obtained by the LRD aligner stand to support the studies of vibrio species performed in other studies [8], [9], showing that the proposed aligner can indeed obtain conclusive results from an evolutionary point of view.

## Methods

This section introduces the sequence aligner that work under Local Rank Distance. First, mathematical preliminaries about the rank-based distance measures are discussed. The LRD aligner and several optimization strategies are presented next.

### Preliminaries

Given a string 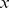 over an alphabet 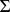, and a character 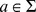, the length of 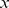 is denoted by 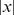, the number of occurrences of the character 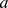 in 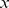 is denoted by 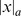. Strings are considered to be indexed starting from position 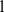, that is 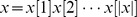. Moreover, 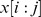 denotes its substring 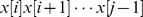.

Given two strings 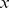 and 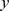 over 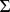, the *rank distance* (RD) between 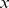 and 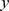, denoted by 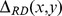, is defined through the following algorithmic process: both strings are scanned (from left to right) and for each character 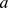 in the first string, and for each of its 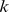-th occurrence in 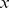 (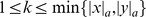), the algorithm sums up the absolute difference between the position of its 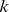-th occurrences in 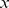 and 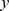. Moreover, for each of the 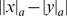 non-matched occurrences of 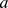 in one of the two strings, the algorithm adds to the sum the arithmetic mean of 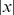 and 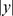, as described in ([30], Definition 2). The total sum computed by this algorithm represents the rank distance. Rank distance [3] is a low computational complexity measure of similarity with various applications in computational biology, from phylogenetic analysis [30], [31] to finding common patterns in DNA sequences [32].

A recently introduced distance measure, termed Local Rank Distance [2], comes from the idea of better adapting rank distance to string data, in order to capture a better similarity (or dissimilarity) between strings, such as DNA sequences or text. Local Rank Distance (LRD) has already shown promising results in computational biology [2] and native language identification [4].

Local Rank Distance is inspired by rank distance, the main differences being that it uses 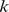-mers instead of single characters, and that it matches each 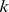-mer in the first string with the nearest equal 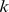-mer in the second string. Given a fixed integer 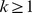, a threshold 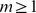, and two strings 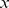 and 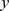 over 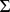, the *Local Rank Distance* between 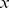 and 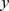, denoted by 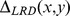, is defined through the following algorithmic process. For each position 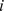 in 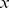 (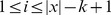), the algorithm searches for that position 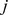 in 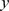 (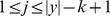) such that 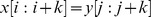 and 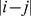 is minimized. If 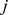 exists and 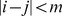, then the offset 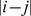 is added to the Local Rank Distance. Otherwise, the maximal offset 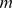 is added to the Local Rank Distance. An important remark is that LRD does not impose any mathematically developed global constraints, such as matching the 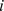-th occurrence of a 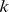-mer in 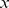 with the 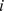-th occurrence of that same 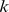-mer in 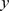. Instead, it is focused on the local phenomenon, and tries to pair equal 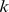-mers at a minimum offset. To ensure that LRD is a (symmetric) distance function, the algorithm also has to sum up the offsets obtained from the above process by exchanging 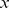 and 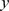. LRD can be formally defined as follows.

**Definition 1** *Let* 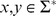 *be two strings, and let* 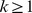 *and* 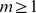 *be two fixed integer values. The Local Rank Distance between* 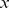 *and* 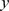 *is defined as*: 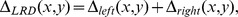

*where* 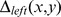 *and* 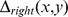 *are defined as follows:*

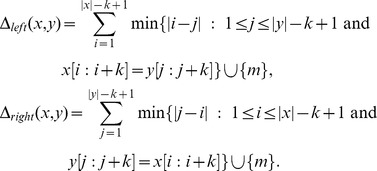



Notice that in order to be a symmetric distance measure, LRD must consider every 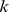-mer in both strings. The symmetric property of LRD is ensured by computing both 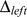 and 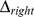. It is easy to observe that 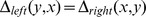. An interesting remark is that overlapping 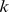-mers are permitted in the computation of LRD, since there is no restriction that tells where 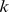-mers should start or end in a DNA string. Another interesting remark is that the search for matching 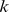-mers is limited within a window of fixed size. The size of this window is determined by the maximum offset parameter 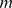. This parameter must be set a priori and should be proportional to the size of the alphabet, the 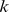-mers, and to the lengths of the DNA strings. Finding similar matches beyond this window is costly and it may also bring unwanted noise in the process. More details about the setting up the parameters of LRD are given in [2].

To better understand how LRD actually works, it is useful to consider the following example where LRD is computed between two strings using 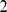-mers.

**Example 1** *Given two strings* 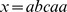 *and* 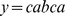, *a fixed maximal offset* 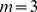, *and a fixed size of* 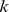 *-mers* 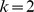, 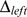 *and* 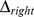 *are computed as follows*: 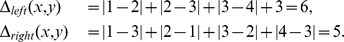



*By summing up the two partial sums, Local Rank Distance is obtained*

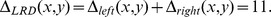



### LRD Aligner

The aligner proposed in this paper is based on Local Rank Distance. It aligns a read of length 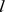 against a reference DNA sequence of length 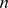. For efficiency reasons, it actually computes only 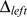 from Definition 1 between the read and a certain substring from the reference genome. It is perfectly reasonable to use only one of the two partial sums, 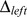 or 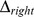, since the symmetric property of LRD is no longer needed in the context of sequence alignment.

The basic alignment algorithm compares the read with the first substring in the reference and remembers the offset of each 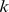-mer in the read. As it continues to compare the read with the following substrings at position 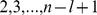 in the reference genome, respectively, the algorithm only needs to update the offset of each 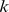-mer to obtain the new 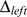 distance at a certain position. The read is aligned in the position that gives the minimum 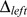 distance, but only if the obtained distance is less than a certain threshold. This basic LRD aligner is provided in the software package. It is worth mentioning that the algorithm described above is also applied for the short DNA string obtained by reverse complementing the original read. Several efficiency improvements are described next. In the end, they lead to the development of the faster LRD aligner presented in Table 12.

**Table 12:** Algorithm 1. The hash LRD aligner algorithm.

**Input:**
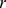 – a short DNA string of length 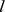 ;
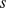 – a reference DNA sequence of length 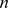 ;
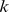 – the size of the 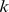 -mers to be compared;
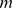 – the maximum offset;
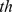 – the maximum rank distance threshold accepted for the aligned read;
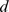 – the threshold that can be adjusted to skip the alignment at some positions.
**Initialization:**
1. 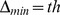 ;
2. 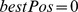 ;
**Computation:**
3. **for** 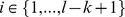
4. add 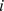 in the array stored at 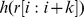 ;
5. **for** 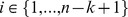
6. **if** 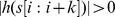 **then** 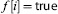 ;
7. **else** 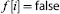 ;
8. 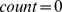 ;
9. **for** 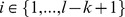
10. **if** 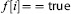 **then** 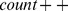 ;
11. 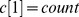 ;
12. **for** 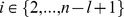
13. **if** 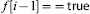 **then** 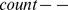 ;
14. **if** 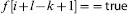 **then** 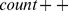 ;
15. 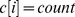 ;
16. **for** 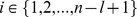
17. **if** 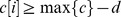 **and** 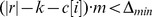 **then**
18. 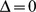 ;
19. **for** 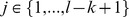
20. **if** 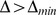 **then**
21. abort and proceed to the next value of 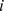 in the loop from step 12;
22. **else**
23. **if** 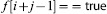 **then**
24. do a binary search in the array stored at 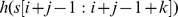
to obtain the position 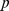 that minimizes 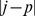
25. 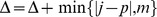 ;
26. **else**
27. 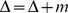 ;
28. **if** 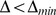 **then**
30. 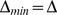 ;
31. 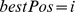 ;
**Output:**
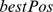 – the position were the read 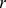 was aligned;
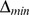 – the minimum LRD (or 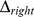 to be more precise) obtained at position 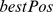 .

#### Indexing Strategies and Efficiency Improvements

The main efficiency improvement brought to the LRD aligner is to store 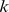-mer positions in a hash table for each read. More precisely, the hash table 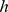 constructed from a short DNA read 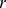 will contain an array for each 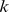-mer in the read. The array will contain all the positions of that 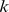-mer in the read 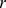. This hash table is actually a positional inverted index structure that is very popular in information retrieval. When LRD is computed for the read at a certain position 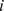 in the reference genome 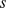, it is no longer necessary to do an extensive search within a window of fixed size to find equal 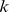-mers between the read and the substring 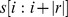. The alternative solution is to take every 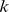-mer in 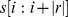 and to look it up in the hash table 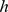. Let 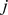 denote the position of the currently considered 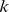-mer in 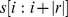. If the 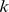-mer is found in 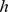, the next step is to try a binary search in the positional array that is stored in 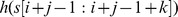, in order to find the nearest position 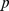 that minimizes 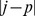. The offset 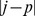 is added to the distance sum only if 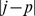 is less than the maximal offset 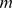, otherwise, 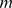 is added. If the 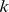-mer is not found in 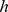, 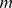 is added to the distance sum. The final sum obtained by this algorithm is the 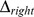 partial sum from Definition 1. As mentioned before, one of the two partial sums can be left out for efficiency reasons, without affecting the accuracy. Thus, the hash LRD implementation is based only on 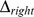, as opposed to the basic implementation that uses only 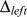. Consequently, there are some minor differences in the results obtained by the two implementations, but the accuracy levels are very similar and in some cases almost the same. For instance, in the experiments performed to evaluate the aligners in the presence of contaminated reads, the hash LRD aligner is only slightly better, while for the task of clustering unknown organisms, the results of the two LRD aligners are exactly the same.

The following strategies are designed to further improve the hash LRD aligner, in terms of speed. First of all, a boolean array 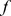 of size 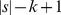 is used. Each element 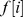 indicates if the 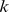-mer 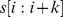 is in the hash table 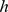. When the algorithm tries to align the read at every position 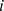 in the reference sequence 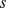, by computing the distance from the read 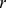 to the substring 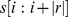, it will have to look up some of the 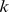-mers in 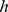, several times (more precisely, 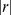 times). Despite the fact that the hash table look up takes 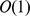 time in theory, it is still faster to check the value of 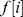 instead of doing a hash table look up. Another improvement is to stop the alignment at a certain position 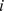, if the distance sum computed between 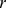 and 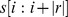 becomes greater than the minimum 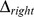 obtained so far.

The next efficiency improvement is to count the number of 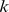-mers that are found in 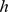, for every substring 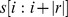 in the reference genome. These counts are stored in an array 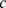 of length 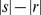. The algorithm can now consider the alignment only in the positions in the reference that are more likely to give the minimum 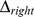 distance. It is fairly easy to observe that the more equal 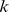-mers 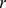 and 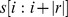 have in common, the lower 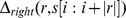 should be, since LRD is first based on finding equal 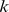-mers between the two strings and, then, on minimizing the offsets between these 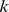-mers. However, there is no guarantee that this is always the case. Therefore, the approach to skip the alignment for some positions 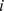 with low 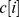, in order to speed up the hash LRD aligner, gives approximate alignment results. More precisely, lower distances can probably be obtained for some of the disregarded positions. These positions are disregarded by the two rules described next. The first rule is to eliminate the position 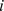 if 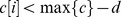, where 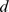 is a new input parameter of the aligner. This parameter can take values in the interval 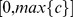. When 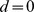, more positions are disregarded. When 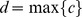, no positions are disregarded at all, since 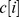 is always greater than 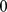. If the parameter 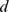 is set to eliminate more positions during the alignment, the algorithm will be faster, but it will also give less accurate alignment results. However, choosing 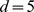 for reads of length 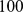 gives similar results to the basic LRD aligner in terms of accuracy, while drastically reducing the computational time, as the empirical results presented in this paper show. In all the experiments, the results of the approximate hash LRD aligner are obtained with 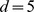. The second rule used by the approximate aligner is to eliminate the position 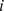, if 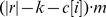 is greater than the minimum 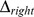 distance obtained until position 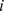. The difference 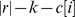 gives the number of 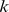-mers in 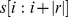 that are not found in 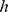. For each of these missing 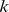-mers, the maximal offset 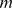 is added to the 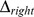 sum. Thus, 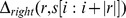 is always greater than 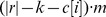. But, if 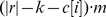 is already greater than the minimum 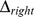 distance obtained so far, there is no point in aligning the read at position 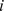.

All the improvements described above are actually combined together to obtain an efficient LRD aligner. It is fairly obvious that these efficiency improvements and indexing strategies produce a different yet more efficient algorithm than the basic LRD aligner. The approximate hash LRD aligner algorithm is described in Table 12. As for the basic LRD aligner, a read is only aligned if the minimum LRD (or 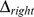, to be more precise) obtained by the algorithm is less than a certain threshold.

The algorithm described in Table 12 is also applied for the short DNA string obtained by reverse complementing the original read. But, another speed improvement is considered here. The alignment tool tries to align the reverse complement only if the minimum distance for the original read is not acceptable. An internal threshold is used to determine if the minimum 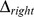 is acceptable (lower than the threshold) or not. This threshold is computed as follows: 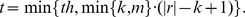



The threshold 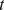 is low enough to ensure, with a certain probability, that if 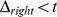 then the read is aligned in the right place. This parameter speeds up the alignment tool especially when the reads and the reference genome belong to the same species. If the reads belong to other species (as in the case of contaminated reads, for example), the aligner will most likely try to align the reverse complements too.

Finally, the computational complexity of the algorithm described in Table 12 is 
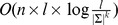
. Unlike the basic LRD aligner, the computational time of the approximate hash LRD aligner is no longer limited by the maximal offset 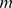 of LRD. This is a clear advantage of this faster implementation. However, in the experiments, the results of both the basic LRD aligner and the approximate hash LRD aligner are obtained with 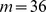 in order to compare the results of the two aligners and to show that they produce almost the same results.

In practice, the input parameters of the algorithm described in Table 12 should be carefully adjusted with respect to length of the DNA reads and to the amount of mutations and errors in DNA. For example, setting 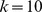 to use 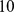-mers for reads of 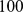 or 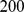 bases is not reasonable, since finding similar 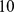-mers in such short DNA strings is rare, if not almost impossible. But 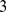 to 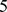-mers are probably more suitable for aligning short DNA reads. Notice that the maximum offset parameter 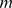 should be adjusted accordingly. Using 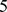-mers and a maximum offset that is too small (less than 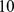, for example) might result in finding almost no similar 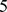-mers in the search window. The best practice to choose the parameters of the aligner is to tune them on a validation data set first.
